# Lymph node yield and tumour subsite are associated with survival in stage I–III colon cancer: results from a national cohort study

**DOI:** 10.1186/s12957-019-1604-x

**Published:** 2019-04-02

**Authors:** Jakob Lykke, Jacob Rosenberg, Per Jess, Ole Roikjaer, On behalf of the Danish Colorectal Cancer Group

**Affiliations:** 1 Department of Surgery, Herlev Hospital, University of Copenhagen, Herlev Ringvej 75, 2730 Herlev, Denmark; 2 Department of Surgery, Roskilde Hospital, University of Copenhagen, Sygehusvej 10, 4000 Roskilde, Denmark

**Keywords:** Colon cancer, Lymph node yield, Tumour subsite, Survival

## Abstract

**Background:**

It has been suggested that apart from tumour and nodal status, a range of patient-related and histopathological factors including lymph node yield and tumour location seems to have prognostic implications in stage I–III colon cancer. We analysed the prognostic implication of lymph node yield and tumour subsite in stage I–III colon cancer.

**Methods:**

Data on patients with stage I to III adenocarcinoma of the colon and treated by curative resection in the period from 2003 to 2011 were extracted from the Danish Colorectal Cancer Group database, merged with information from the Danish National Patient Register and analysed.

**Results:**

A total of 13,766 patients were included in the analysis. The 5-year overall survival ranged from 59.3% (95% CI 55.7–62.9%) (lymph node yield 0–5) to 74.0% (95% CI 71.8–76.2%) (lymph node yield ≥ 18) for patients with stage I–II disease (*p* < 0.0001) and from 36.4% (95% CI 29.8–43.0%) (lymph node yield 0–5) to 59.4% (95% CI 56.6–62.2%) (lymph node yield ≥ 18) for patients with stage III disease (*p* < 0.0001). The 5-year overall survival for tumour side left/right was 59.3% (95% CI 57.9–60.7%)/64.8% (CI 63.4–66.2%) (*p* < 0.0001). In the seven colonic tumour subsites, the 5-year overall survival ranged from 56.6% (95% CI 51.8–61.4%) at splenic flexure to 65.8% (95% CI 64.5–67.2%) in the sigmoid colon (*p* < 0.0001). In a cox regression analysis, lymph node yield and tumour side right/left were found to be prognostic factors. Tumours at the hepatic and splenic flexures had an adverse prognostic outcome.

**Conclusion:**

For stage I–III colon cancer, a lymph node yield beyond the recommended 12 lymph nodes was associated with improved survival. Both subsite in the right colon, as well as subsite in the left colon, turned out with adverse prognostic outcome questioning a simple classification into right-sided and left-sided colon cancer.

## Introduction

The TNM Classification of Malignant Tumours (TNM) proposed by the American Joint Committee on Cancer [1] is the most widely used staging system. Moreover, subsequent studies have demonstrated that, in non-metastatic colon cancer, apart from T and N category, a range of patient- and tumour-related factors including tumour location and lymph node yield seems to be associated with survival in stage I–III colon cancer. “The possible association between lymph node yield and survival is debated: On the one side it has been argued that a high lymph node yield, per se, improves survival, whereas, on the other side, it has been argued that a high lymph node yield reduces the stage drifting effect and thereby enhances survival” [2]. Furthermore, it has been proposed that cancers in the colon differ according to the tumour subsites, which is also reflected in survival [3–8]. Nevertheless, the association between lymph node yield, tumour subsites and survival in colon cancer has not previously been exclusively assessed in an extensive national cohort study.

The aim of this nationwide study was, based on prospectively collected data from a national cohort of patients with stage I–III radically resected colon cancer, to examine the prognostic implications of patient and tumour-related factors including lymph node yield and tumour subsite and in colon cancer.

## Methods

This nationwide cohort study is a result of data derived from three Danish registers: the Danish Colorectal Cancer Group (DCCG), the Danish Pathology Registry and the Danish National Patient Registry.

Patients with a first-time diagnosis of colonic stage I–III adenocarcinoma subjected to curative resection between May 2001 and December 2011 were identified in the DCCG database, described in details elsewhere by this group [9]. Briefly, since May 2001, the DCCG database, a subgroup of the Danish Surgical Society, has included all Danish patients with a first-time diagnosis of colon carcinoma. Surgery for colon cancer is performed only at public hospitals in Denmark. The surgical departments provide the data for the DCCG database. The database has at data completeness of more than 95% [10]. Histopathology of the tumour was extracted from the Danish Pathology Registry.

The cohort extracted from the DCCG database was merged with data from the Danish National Patient Register, using the unique personal identification number given to all Danish citizens. Confounders possibly associated with survival were extracted from the DCCG database for use in the present study (Table 1). The Dukes classification was standard for staging patients with colorectal cancer in Denmark in the first 2 years of the database (2001–2002) [11], but since the Dukes classification is not specific about the pT stage, we decided to exclude patients from that period. All patients in the period 1 Jan 2003 to 31 December 2011 with a first-time diagnosis of adenocarcinoma of the colon with a subsequent R0 resection of a stage I–III cancer were included. The border between the sigmoid colon and the rectum was defined as 15 cm beyond the anal verge.

**Table 1:** Patient and tumour characteristics

*n* = 13,766	*n* (%)
Gender	
Male	6694 (48.6)
Female	7072 (51.4)
Age (years)	
< 65	2011 (14.6)
65–75	6528 (47.4)
> 75	5227 (38.0)
Location	
Right	7265 (52.8)
Left	6501 (47.2)
Location, tumor subsite	
Coecum	2718 (19.7)
Ascending colon	1859 (13.5)
Hepatic flexure	993 (7.2)
Transverse colon	1104 (8.0)
Splenic flexure	591 (4.3)
Descending colon	580 (4.2)
Sigmoid colon	5921 (43.0)
T stage	
pT1	728 (5.3)
pT2	1566 (11.4)
pT3	9078 (65.9)
pT4	2303 (16.7)
Missing value	91 (0.7)
N stage	
N0	8607 (62.5)
N1	3232 (23.5)
N2	1927 (14.0)
Priority of surgery	
Elective	11,918 (86.6)
Acute	1845 (13.4)
Missing value	3 (0.0)
Type of surgery	
Open	9683 (70.3)
Laparoscopic	4080 (29.6)
Missing value	3 (0.0)
Blood transfusion	
Transfusion	3140 (22.8)
No transfusion	10,036 ( 72.9)
Missing value	590 (4.3)
Postoperative anastomotic leak	
No leak Leak Missing value	12,432 (90.3) 744 (5.4) 590 (4.3)
Total lymph node yield	
Median (quartiles)	15 (11–22)
Lymph node yield	
0–5	1001 (7.3)
6–11	2938 (21.3)
12–17	4439 (32.2)
≥ 18	5388 (39.1)
ASA score	
ASA I	2673 (20.3)
ASA II	7280 (55.3)
ASA III	2981 (22.6)
ASA IV	233 (1.8)
Missing value	9 (0.0)
Charlson score	
1	8349 (60.6)
2	1730 (12.6)
3	720 (5.2)
4	2377 (17.3)
Missing value	590 (4.3)

*ASA* American Society of Anaesthesiologists

Data were statistically analysed using the IBM SPSS version 22 (IBM Corp., Armonk, NY, USA). The patient characteristics and tumour-related data were described by non-parametric statistics. Overall survival was analysed according to sex, age, acute vs elective surgery, open vs laparoscopic surgery, American Society of Anaesthesiologists (ASA) score, Charlson comorbidity index, blood transfusion, postoperative anastomotic leakage, year of diagnosis, pT category and in four groups defined by the lymph node yield and by the tumour localisation. The localisation of the colon cancer was treated both according to the categorisation into right-sided colon cancer (coecum, ascending colon, hepatic flexure or transverse colon) and left-sided colon cancer (from the splenic flexure to the sigmoid colon, both included) and to the specific subsites of the colon. Moreover, overall survival was analysed for the lymph node yield groups after stratifying for lymph node-positive disease (stage III) versus lymph node-negative disease (stage I to II). Kaplan-Meier plots and log-rank tests were used for survival analysis. The association between sex, age, acute vs elective surgery, open vs laparoscopic surgery, ASA score, Charlson comorbidity index, blood transfusion, postoperative anastomotic leakage, year of diagnosis, T stage and the four groups defined by the lymph node yield and by the tumour localisation was explored using multiple logistic regressions with overall survival as outcome measure and reported as hazard ratios (HR) with 95% confidence intervals (95% CI). Thus, lymph node yield and pT stage at each level of the variable were compared with the preceding one. Since this group has previously described that year of diagnosis was related to the lymph node yield for both colon [9] and rectal cancer [12], most likely because the treatment of colon and rectal cancer in Denmark has been centralised and standardised throughout the study period [13], year of diagnosis was chosen for further adjustment. A *p* value of < 0.05 was defined as the level of significance in all of the analyses.

## Results

A number of 13,766 patients (48.7% males) with an R0 resection of UICC stage I–III first-time diagnosis of colonic cancer were available for our analysis. The median age was 70 years (interquartile range (IQR) 62–78), and the median lymph node yield was 15 (IQR 11–22). A number of 5159 (37.5%) patients had lymph node-positive disease (Table 1).

**Fig. 1:**
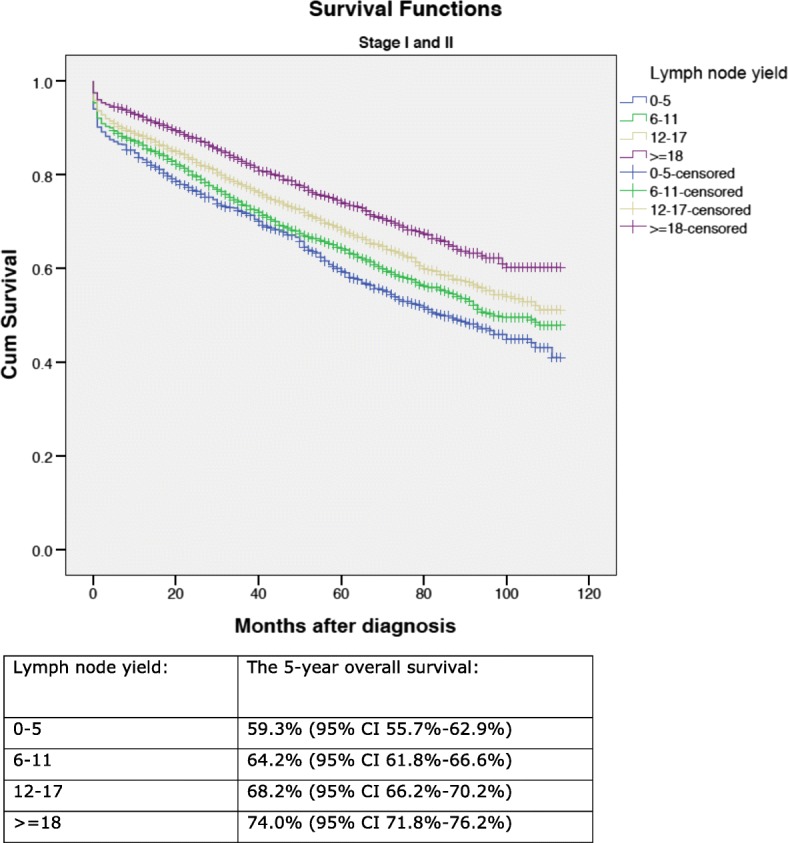
Overall 5-year survival according to lymph node yields in the group of patients with stage I–II colon cancer

**Fig. 2:**
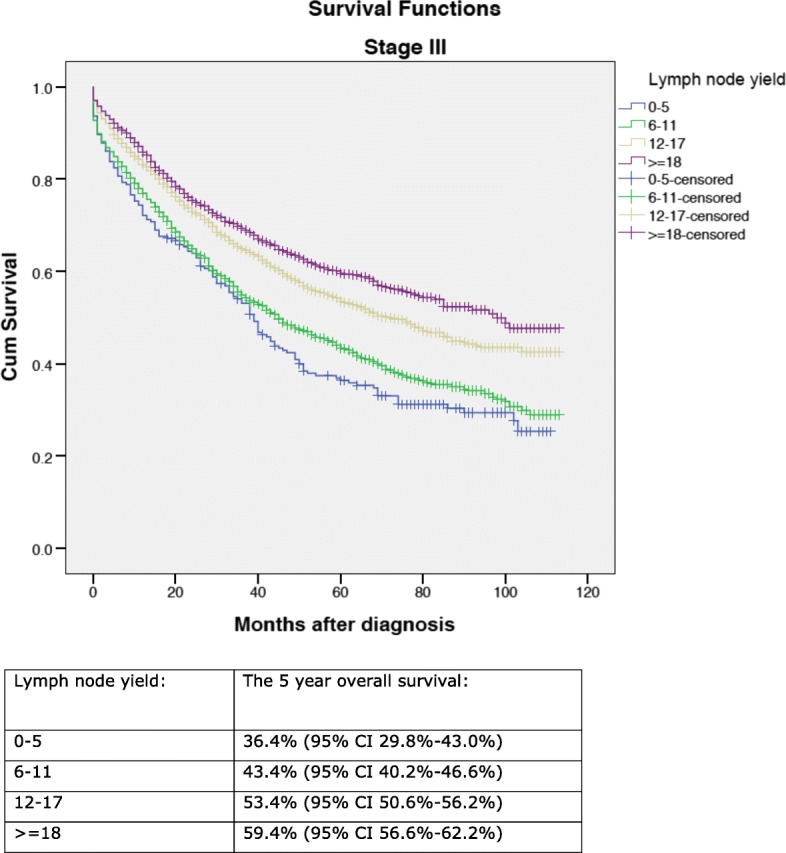
Overall 5-year survival according to LNY in the group of patients with stage III colon cancer

In the entire cohort, the 5-year overall survival was 62.2% (95% CI 61.2–63.2%). In patients with lymph node-negative (stage I–II) disease, the 5-year overall survival according to the lymph node yield was 59.3% (95% CI 55.7–62.9%) (lymph node yield 0–5), 64.2% (95% CI 61.8–66.6%) (lymph node yield 6–11), 68.2% (95% CI 66.2–70.2%) (lymph node yield 12–17) and 74.0% (95% CI 71.8–76.2%) (lymph node yield ≥ 18), (*p* < 0.0001) (Fig. 1). In patients with lymph node-positive (stage III) disease, the 5-year overall survival according to the lymph node yield was 36.4% (95% CI 29.8–43.0%) (lymph node yield 0–5), 43.4% (95% CI 40.2–46.6%) (lymph node yield 6–11), 53.4% (95% CI 50.6–56.2%) (lymph node yield 12–17) and 59.4% (95% CI 56.6–62.2%) (lymph node yield ≥ 18), (*p* < 0.0001) (Fig. 2).

The 5-year overall survival in right-sided colon cancer and left-sided colon cancer was 59.3% (95% CI 57.9–60.7%) and 64.8% (95% CI 63.4–66.2%), respectively, (*p* < 0.0001).

The 5-year overall survival for patients with cancer in the sigmoid colon was 65.8% (95% CI 64.5–67.2%), descending colon 63.3% (95% CI 58.5–68.1%), splenic flexure 56.6% (95% CI 51.8–61.4%), transverse colon 57.7% (95% CI 54.3–61.1%), hepatic flexure 57.4% (95% CI 53.8–61.2%), ascending colon 62.4% (95% CI 59.8–65.0%) and caecum 58.6% (95% CI 56.4–60.8%), (*p* < 0.0001).

In the Cox regression analysis including gender, age, pN category, pT category, the priority of surgery, anastomotic leak, blood transfusion, Charlson score, ASA score, year of diagnosis, lymph node yield, tumour subsite and tumour side left/right, we found all of the variables to be independent prognostic factors. With regard to tumour subsite, we only found that tumours at the hepatic and splenic flexures had an adverse prognostic association. The details are given in Table 2.

**Table 2:** Cox proportional hazards regression analysis including (a) tumour side left/right and (b) tumour subsite

Variable	Hazard ratio (95% CI)	*p* value
a. Tumour side left/right		
Male	1	
Female	0.819 (0.771–0.869)	< 0.0001
ASA I compared to ASA II	0.728 (0.658–0.806)	< 0.0001*
ASA II compared to ASA III	0.613 (0.573–0.655)	< 0.0001*
ASA III compared to ASA IV	0.646 (0.552–0.756)	< 0.0001*
Laparoscopic surgery	1	
Open surgery	1.127 (1.033–1.230)	0.007
No blood transfusion	1	
Blood transfusion	1.315 (1.232–1.403)	< 0.0001
Age 65 years compared to 65–75 years	0.693 (0.615–0.781)	< 0.0001*
Age 65 years compared to age > 75 years	0.504 (0.472–0.537)	< 0.0001*
Year of diagnosis	0.967 (0.952–0.983)	< 0.0001*
LNY 0–5 compared to LNY 5–11	1.135 (1.019–1.264)	0.021*
LNY 5–11 compared to LNY 12–17	1.240 (1.148–1.340)	< 0.0001*
LNY 12–17 compared to LNY ≥ 18	1.139 (1.054–1.230)	< 0.001*
N0	1	
N1 + 2	0.609 (0.558–0.665)	< 0.0001
pT1 compared to pT2	0.817 (0.673–0.992)	0.041*
pT2 compared to pT3	0.824 (0.739–0.917)	< 0.0001*
pT3 compared to pT4	0.614 (0.571–0.661)	< 0.0001*
Tumour side left	1	
Tumour side right	1.069 (1.005–1.136)	0.033
Charlson score 0 compared to score 1	0.792 (0.725–0.864)	< 0.0001
Charlson score 1 compared to score 2	0.908 (0.793–1.040)	0.165
Charlson score 2 compared to score 3	0.835 (0.735–0.948)	0.005
No leak	1	
Leak	1.759 (1.575–1.965)	< 0.0001
Elective surgery	1	
Acute surgery	1.756 (1.628–1.893)	< 0.0001
b. Tumour subsite		
Male	1	
Female	0.820 (0.772–0.870)	< 0.0001
ASA I compared to ASA II	0.729 (0.659–0.806)	< 0.0001*
ASA II compared to ASA III	0.613 (0.573–0.656)	< 0.0001*
ASA III compared to ASA IV	0.646 (0.552–0.756)	< 0.0001*
Laparoscopic surgery	1	
Open surgery	1.120 (1.026–1.233)	0.007
No blood transfusion	1	
Blood transfusion	1.309 (1.226–1.397)	< 0.0001
Age 65 years compared to age 65–75 years	0.691 (0.614–0.779)	< 0.0001*
Age 65 years compared to age > 75 years	0.503 (0.471–0.536)	< 0.0001*
Year of diagnosis	0.967 (0.951–0.982)	< 0.0001*
LNY 0–5 compared to LNY 5–11	1.134 (1.018–1.262)	0.023*
LNY 5–11 compared to LNY 12–17	1.240 (1.147–1.339)	< 0.0001*
LNY 12–17 compared to LNY ≥ 18	1.142 (1.057–1.234)	< 0.001*
N0	1	
N1 + 2	0.606 (0.555–0.663)	< 0.0001
pT1 compared to pT2	0.818 (0.674–0.993)	0.042*
pT2 compared to pT3	0.825 (0.740–0.919)	< 0.0001*
pT3 compared to pT4	0.614 (0.571–0.660)	< 0.0001*
Sigmoid colon	1	
Coecum	1.077 (0.993–1.168)	0.074
Ascending colon	1.032 (0.937–1.137)	0.519
Hepatic flexure	1.213 (1.084–1.358)	< 0.001
Transverse colon	1.058 (0.948–1.180)	0.315
Splenic flexure	1.181 (1.026–1.359)	0.021
Descending colon	0.945 (0.813–1.109)	0.514
Charlson score 0 compared to score 1	0.792 (0.725–0.864)	< 0.0001*
Charlson score 1 compared to score 2	0.908 (0.793–1.040)	0.165*
Charlson score 2 compared to score 3	0.835 (0.735–0.948)	0.005*
No leak	1	
Leak	1.753 (1.569–1.960)	< 0.0001
Elective surgery	1	
Acute surgery	1.753 (1.626–1.891)	< 0.0001

*ASA* American Society of Anaesthesiologists, *LNY* lymph node yield

*Each level compared to the preceding level

## Discussion

In this national cohort of prospectively collected data of more than 13,000 patients with colon cancer, we have demonstrated a significant association between lymph node yield, tumour subsite and overall survival in stage I–III colon cancer irrespectively of the N and T category.

Several mixed studies of colon and rectal cancer have indicated an association between the lymph node yield and survival, especially for stage II and III disease [14–18]. This is supported by our results, but so far, the exact oncological explanation for this observation is unknown. As previously discussed by this group [17], a possible explanation could be that a high lymph node yield reduces the risk of under staging by increasing the chance of identification of patients with stage III disease [19]. In patients with a low lymph node yield, metastatic nodes may be unnoticed resulting in a false negative staging by classifying node-positive disease as node-negative disease. Still, this does not sufficiently explain why patients with node-positive disease with a high lymph node yield have a significantly better overall survival than those with a low lymph node yield.

In our study, we found that a lymph node yield exceeding the recommended minimum of 12 lymph nodes was independently associated with improved survival for stage III as well as stage I–II disease. A part of the explanation could be that a high lymph node yield is a surrogate marker for the quality of surgery, e.g. as in complete mesocolic excision with correct central ligation [20–22], but unfortunately, such data were not available for the analysis.

Another part of the explanation could be that the immunological interaction between the tumour and the host may influence the number of detectable lymph nodes, and it has been proposed that a smaller lymph node yield represents a weakened immune response leading to smaller lymph nodes that are more difficult to identify in the surgical specimen [23, 24].

It has previously been suggested that carcinomas of the right and left colon should be considered as different tumour entities [5]. Thus, it has been demonstrated that right-sided and left-sided colon cancers are significantly different regarding epidemiology, clinical course and prognosis [6, 7]. Moreover, it has been proposed that these discrepancies may be caused by genetic differences that account for distinct carcinogenesis and biological behaviour [3, 4, 25]. Nevertheless, this categorisation into right-sided and left-sided colon cancer based on the embryological origin has been questioned in recent studies, where the carcinoma of the colon has been analysed according to tumour subsite [26, 27].

Even though we have found a significant difference in tumour side right vs left, our results also question this dichotomy into right-sided and left-sided colon cancer according to survival, since we found that only tumours at the hepatic and splenic flexure were associated with an independently adverse prognosis compared to tumours at the sigmoid colon. No difference in survival between tumours in the rest of the tumour subsites and tumours at the sigmoid colon were observed. The reason for that is unknown, and one can only speculate whether the association between adverse independent prognostic outcome and tumours at the hepatic and splenic flexure is due to operational difficulties, including central ligation of the branches of the middle colic artery or specific immunology of tumours at these sites.

An increase in the 5-year overall survival during the period of data collection was observed. Thus, in the multivariate analysis, the year of diagnosis was an independent prognostic factor. So far the explanation for that finding is unknown. However, in Denmark, there has been an increased focus over the last 15 years on colorectal cancer including national guidelines and programs for diagnosis and treatment of colon and rectal cancer [13, 28]. It is liable that this increased focus, including improvements in diagnosing colon cancer, has contributed to the observed increase in survival.

The present study was the strength by the inclusion of patients from all Danish departments conducting colon surgery during the study period and was further strengthened by data merged from two different population-based national registers.

There are some limitations to our study: Firstly, since it is an observational study, we did not have the possibility to prove causality. Secondly, since no data on cancer recurrence had been registered in our dataset, we were only able to compare 5-year overall survival and not cancer-free survival.

Lack of information on chemotherapy is also a potential limitation of the study, since chemotherapy may have prognostic implications. In the present study, it was not possible to differentiate between patients receiving adjuvant chemotherapy and those who were not. Since 1997, adjuvant chemotherapy (5-FU + leucovorin) has been the standard treatment for stage III colon cancer patients, and since 2009, adjuvant chemotherapy (5-FU ± oxaliplatin) has been offered for selected stage II high-risk patients in Denmark [28].

Finally, no genetic or biological data were available in our dataset leaving us without the possibility to further qualify the observed differences between the tumour subsites.

## Conclusion

Our results demonstrate that the total number of lymph nodes harvested is related to survival in stage I–II and III colon cancer. Stage migration might be a part of the explanation for the prognostic implications of a high lymph node yield, but this is only a part of the explanation since we have demonstrated that a lymph node yield beyond the recommended 12 lymph nodes was associated with improved survival in stage I–II and stage III disease. “Good surgery” with correct mesocolic dissection and true central ligations as well as the immunology of the tumour and host could also be a part of the explanation.

Even though we found a significant difference in survival in right- vs left-sided colon cancer, our results support that a classification of colon cancer into right-sided and left-sided disease does not represent the entire complexity of this tumour since both subsites in the right colon, as well as subsites in the left colon, turned out with adverse prognostic outcome. Further research should consider whether tumour subsite should be taken into account when the strategies for diagnosis and treatment of colon cancer are decided.

## References

[1] Edge SB, Compton CC (2010). The American Joint Committee on Cancer: the 7th edition of the AJCC cancer staging manual and the future of TNM. Ann Surg Oncol.

[2] Resch A, Langner C (2013). Lymph node staging in colorectal cancer: old controversies and recent advances. World J Gastroenterol.

[3] Benedix F, Kube R, Meyer F, Schmidt U, Gastinger I, Lippert H (2010). Comparison of 17,641 patients with right- and left-sided colon cancer: differences in epidemiology, perioperative course, histology, and survival. Dis Colon Rectum.

[4] Hansen IO, Jess P (2012). Possible better long-term survival in left versus right-sided colon cancer - a systematic review. Dan Med J.

[5] Nawa T, Kato J, Kawamoto H (2008). Differences between right- and left-sided colon cancer in patient characteristics, cancer morphology and histology. J Gastroenterol Hepatol.

[6] Tentes AAK, Korakianitis OS, Kakolyris S (2010). Differences between right- and left-sided colon carcinomas. J BUON.

[7] Yahagi M, Okabayashi K, Hasegawa H (2015). PTH-289 The poor overall survival of right-sided colon cancer compared with left-sided colon cancers: a systematic review and meta-analysis. Gut.

[8] Bleeker WA, Hayes VM, Karrenbeld A, Hofstra RMW, Hermans J, Buys CHCM, et al. Impact of KRAS and TP53 mutations on survival in patients with left- and right-sided Dukes’ C colon cancer. Am J Gastroenterol [Internet]. 2000;95(10):2953-7 http://www.ncbi.nlm.nih.gov/pubmed/11051374. Accessed 26 Mar 2019. 10.1111/j.1572-0241.2000.02327.x 11051374

[9] Lykke J, Jess P, Roikjær O, Danish Colorectal Cancer Group (2016). A high lymph node yield in colon cancer is associated with age, tumour stage, tumour sub-site and priority of surgery. Results from a prospective national cohort study. Int J Color Dis.

[10] DCCG Annual Report 2011. https://dccg.dk/wp-content/uploads/2017/10/Aarsrapport_2011.pdf. Accessed 27 May 2013.

[11] DCCG Anual Repport 2001-2. https://dccg.dk/wp-content/uploads/2017/10/Aarsrapport_20012002.pdf. Accessed 12 Sept 2014.

[12] Lykke J, Roikjaer O, Jess P. Tumour stage and preoperative chemoradiotherapy influence the lymph node yield in stages I-III rectal cancer results from a prospective nationwide cohort study. Color Dis. 2013. 10.1111/codi.12521. 10.1111/codi.12521 24329928

[13] Lykke J, Roikjær O, Jess P (2013). The majority of surgical departments adhere to national Danish guidelines for surveillance after colorectal cancer surgery. Dan Med J.

[14] Caplin S, Cerottini JP, Bosman FT, Constanda MT, Givel JC (1998). For patients with Dukes’ B (TNM Stage II) colorectal carcinoma, examination of six or fewer lymph nodes is related to poor prognosis. Cancer.

[15] Swanson RS, Compton CC, Stewart AK, Bland KI. The prognosis of T3 N0 colon cancer is dependent on the number of lymph nodes examined. Ann Surg Oncol. 10(1):65–71 http://www.ncbi.nlm.nih.gov/pubmed/12513963. Accessed 24 Mar 2013. 10.1245/aso.2003.03.058 12513963

[16] Chen SL, Bilchik AJ (2006). More extensive nodal dissection improves survival for stages I to III of colon cancer: a population-based study. Ann Surg.

[17] Lykke J, Roikjaer O, Jess P. The relation between lymph node status and survival in stage i-iii colon cancer: results from a prospective nationwide cohort study. Color Dis. 2012. 10.1111/codi.12059. 10.1111/codi.12059 23061638

[18] Peeples C, Shellnut J, Wasvary H, Riggs T, Sacksner J (2010). Predictive factors affecting survival in stage II colorectal cancer: is lymph node harvesting relevant?. DisColon Rectum.

[19] George S, Primrose J, Talbot R (2006). Will Rogers revisited: prospective observational study of survival of 3592 patients with colorectal cancer according to number of nodes examined by pathologists. Br J Cancer.

[20] Bertelsen CA, Neuenschwander AU, Jansen JE (2014). Disease-free survival after complete mesocolic excision compared with conventional colon cancer surgery: a retrospective, population-based study. Lancet Oncol.

[21] Hohenberger W, Weber K, Matzel K, Papadopoulos T, Merkel S (2009). Standardized surgery for colonic cancer: complete mesocolic excision and central ligation--technical notes and outcome. Color Dis.

[22] Eiholm S, Ovesen H (2010). Total mesocolic excision versus traditional resection in right-sided colon cancer - method and increased lymph node harvest. Dan Med Bull.

[23] Lemmens VE, van Lijnschoten I, Janssen-Heijnen ML, Rutten HJ, Verheij CD, Coebergh J-WW (2006). Pathology practice patterns affect lymph node evaluation and outcome of colon cancer: a population-based study. Ann Oncol.

[24] Sarli L, Bader G, Iusco D (2005). Number of lymph nodes examined and prognosis of TNM stage II colorectal cancer. Eur J Cancer.

[25] Bufill JA (1990). Colorectal cancer: evidence for distinct genetic categories based on proximal or distal tumor location. Ann Intern Med.

[26] Jess P, Hansen IO, Gamborg M, Jess T. A nationwide Danish cohort study challenging the categorisation into right-sided and left-sided colon cancer. BMJ Open. 2013;3(5). 10.1136/bmjopen-2013-002608. 10.1136/bmjopen-2013-002608 PMC3657647 23793665

[27] Benedix F, Meyer F, Kube R (2012). Influence of anatomical subsite on the incidence of microsatellite instability, and KRAS and BRAF mutation rates in patients with colon carcinoma. Pathol Res Pract.

[28] Iversen LH, Green A, Ingeholm P, Østerlind K, Gögenur I (2016). Improved survival of colorectal cancer in Denmark during 2001-2012 - the efforts of several national initiatives. Acta Oncol.

